# Surgical Approach of Oropharyngeal Squamous Cell Carcinoma (SCC) With Transoral Robotic Surgery Versus Non-robotic Transoral Surgery and Laser: A Systematic Review

**DOI:** 10.7759/cureus.81074

**Published:** 2025-03-24

**Authors:** Mohammed Yousif, Lakis Liloglou, Axel Kaehne, Sadia Khawaja, Abduraheem Mohamed, Sundus Mohamed, Ahmed Gadoura, Suliman Ali, Etienne Villeneuve, Mohammed A Elkrim

**Affiliations:** 1 Department of Surgery and Otolaryngology, University Hospital of Manchester Foundation Trust, Manchester, GBR; 2 Department of Otolaryngology, Leicester Royal Infirmary, Leicester, GBR; 3 Faculty of Health, Social Sciences and Medicine, Edge Hill university, Lancashire, GBR; 4 Faculty of Health, Social Sciences and Medicine, Edge Hill University, Lancashire, GBR; 5 General Surgery, Our Lady of Lourdes Hospital, Drogheda, Drogheda, IRL

**Keywords:** functional outcomes, human papillomavirus, non-robotic transoral surgery, ontological outcomes, oropharyngeal squamous cell carcinoma, transoral robotic surgery

## Abstract

Oropharyngeal squamous cell carcinoma (OPSCC) is among the most common head and neck cancers, with increasing use of surgical treatment for stage T1/T2 cases. This study compares the oncological outcomes of transoral robotic surgery (TORS) with non-robotic transoral surgery. A systematic review of the Ovid database was conducted using defined inclusion and exclusion criteria. Data extraction was performed independently by two authors, and publication quality was assessed using the Oxford Centre for Evidence-Based Medicine criteria. Outcomes related to oncological margins, swallowing, and voice function were analyzed descriptively.

Ten studies met the inclusion criteria, most of which were retrospective cohort studies. The comparative analysis demonstrated that TORS provides better overall oncological outcomes than non-TORS in patients with T1/T2 OPSCC. In addition, TORS was associated with a 30% higher rate of achieving clear surgical margins. While these findings suggest that TORS may be the superior modality for oncological and functional outcomes, the study is limited by the lack of randomized controlled trials and non-randomized treatment assignments. Further prospective randomized trials are necessary to confirm these results and establish clinical practice guidelines.

## Introduction and background

Head and neck squamous cell carcinoma (HNSCC) is the 7th most common cancer worldwide and oropharyngeal squamous cell carcinoma (OPSCC) is among the frequent manifestations of this cancer type [1]. During the past three decades, following the validation of molecular HPV assays [2], research demonstrated a strong relationship between this tumor type and the human papillomavirus (HPV) as the cause in a substantial number of patients, especially among non-smokers [3]. HPV+ OPSCC tumors have a different molecular and clinical behavior to the HPV negative ones. The first are more frequent among younger patients and non-smokers while the latter are associated with more classical aerodigestive cancer risk factors, such as smoking and alcohol consumption, and they respond better to chemoradiotherapy (CRT) treatment [4]. A remaining clinical challenge is that. HPV+ OPSCC patients demonstrate a response to chemoradiation that is better than HPV negative [5,6], therefore pushing the need for new treatments has been advocated.

The recent increased interest in transoral surgical techniques and the advances in instrumentation have led to the study of the role of transoral surgeries in the treatment of these tumors. Transoral microscopic surgeries, either with laser (TML) or without, and transoral robotic surgeries (TORS), are among the approaches currently adopted by many surgeons. Despite the good functional results in terms of tissue preservation of both approaches, they also present disadvantages, such as postoperative pain and long recovery times [7,8].

In the last three decades, there has been a significant increase in the incidence of oropharyngeal SCC, especially the tonsils and tongue base. In the UK alone, the number has doubled over the last decade [9]. Regarding clinical symptoms, the tumor arises from the tonsils in about 60%, although sometimes the tumor may be so small that the only clinical sign of its presence is the enlargement of some of the lymph nodes in the neck. The extent of tumor spread locally or regionally be understood by adopting the classification of AJCC 8 (American Joint Committee on Cancer). Thirty percent is found at the base of the tongue. Also, compared to the tonsils, sometimes the tumor at the bottom of the tongue may be without clinical symptoms and may be discovered during a clinical examination to find out the reasons for the enlargement of the lymph node in the neck [10]. In the case of symptoms, whether in the tonsils or the base of the tongue, they are often in the form of dysphagia, odynophagia, referred otalgia, or altered speech. A workup involves a good history and clinical examination either in the outpatient setting or even examination under anesthesia. Ultrasound is useful when there is obvious lymphadenopathy, where fine needle aspiration can be obtained. For staging and tumor extension and outline, cross-sectional imaging such as CT and MRI scan is mandatory. The role of PET CT scans is limited to unknown primary or roll out distance metastasis [10].

The noticeable increase in the prevalence of HPV+ OPSCC and its association with younger patients presents certain clinical challenges with regard to tissue preservation and functionality post-treatment. In addition, developments in radiotherapy and surgical treatments seem to have contributed significantly to the difference of views between the clinicians as well as the patient’s ability to choose any of the treatment modalities. HPV+ and HPV-OPSCC are currently treated with similar chemoradiation regimens. Currently, it is not completely clear to clinicians which treatment modalities (surgical treatment or CRT) would have led to a better prognosis, especially since there are not enough randomized control trials (RCTs) to augment decision-making. However, there is a general agreement that for the early stages (T1 and T2 according to AJCC 8 classification), primary surgical intervention can be the first line of treatment [11]. The NICE guidelines for oropharyngeal carcinoma stages T1 and T2 suggest providing patients with the option of transoral surgical resection or primary radiotherapy for T1-2 N0 oropharyngeal tumors [12] consider postoperative radiotherapy, with or without concomitant chemotherapy, for T1-2 N0 oropharyngeal tumors if pathologically adverse risk factors have been identified.

Transoral microsurgery with or without laser and TORS are the surgical techniques for OPSCC, which have the advantages of being minimally invasive [13]. There are important practical and methodological differences between these techniques that must be considered when using them in the resection of these tumors. The most significant difference is that while the transoral microsurgery technique dictates that the scope of surgery, to minimize removal of uninvolved tissue adjacent to the tumor, is subject to the specific anatomy of the tumor and thus varies from case to case, TORS depends on the advisability of performing a standard procedure to avoid trans-tumoral resection and attempts to adhere to the principles of surgical resection of the tumor as an en-block resection. However, despite all these slight differences, the two approaches have not been compared systematically, to determine whether one has better oncological or functional outcomes than the other. This systematic review therefore aims to provide a summary of the current available knowledge and comparison between the two methods in terms of achieving clear margins and functional outcomes. The purpose of this study is to conduct a thorough review of both surgical interventions and their impact on the oncological and functional outcome of oropharyngeal tumors in the published literature.

## Review

Methods

Literature Search

A systematic literature review was carried out to review all available relevant data. During the article selection process, the authors followed the recommendations made by the Preferred Reporting Items for Systematic Reviews and Meta-Analyses (PRISMA) [14-16]. All authors independently searched the Ovid search engine, which included the AMED database (from 1985), the Embase database (from 1074), and the Ovid MIDLINE database (from 1946). Searches were conducted and were designed to combine disease-specific terms (oropharyngeal, neoplasm, oropharyngeal cancer, and oropharyngeal SCC), treatment-specific terms for robotic surgery (transoral robotic surgery and TORS), and specific terms for non-robotic surgery (TORS, transoral microsurgery, transoral surgical resection, conventional transoral surgery, transoral laser surgery, and transoral non-robotic surgery [17]. The Ovid operator with ‘AND’ and ‘OR’ is used to combine terms. All searches were conducted between March and November 2022.

Inclusion and Exclusion Criteria

Titles and abstracts were then screened to fit the following inclusion criteria: (1) patients with early-stage oropharyngeal squamous cell carcinoma (OPSCC) such as T1 and T2 where transoral resection with TORS was the primary modality; (2) comparative cohorts that received a primary treatment with non-robotic transoral microscopic surgery; (3) analysis of outcomes including clear margins and functional outcomes such as swallowing and voice; (4) English language.

Studies were excluded if any of the following criteria were met: (1) oropharyngeal cancer other than T1 or T2; (2) studies reporting only one functional outcome; (3) studies with incomplete data.

The initial keyword search yielded a total of 127 studies; after removing duplicates, the remaining studies totaled 68. There were 22 studies included after reviewing the abstract. Only ten studies were deemed relevant after reviewing the abstract.

To minimize the risk of bias throughout the study, two independent researchers will conduct the analysis. In cases of discrepancies, a third researcher will be consulted for an opinion. Some papers lack certain demographic data; however, they were still included because the primary aim of the study is to measure oncological and functional outcomes. This omission can be considered a form of selection bias.

Data Extraction

The quality of eligible studies was assessed using a grading tool developed by the Oxford Centre for Evidence-based Medicine. Other factors considered were the clarity and extent of reported outcome data, the inclusion of relevant patient baseline characteristics, and the presence of selection bias. There were no RCT studies eligible for inclusion. As a result, the evidence in this systematic review is entirely comprised of retrospective and observational cohort studies. The full texts of eligible studies that met the above inclusion criteria were obtained and thoroughly searched. Data on study characteristics was extracted for each text such as author, year of publication, study design, sample size, mean age, male and female number, oncological outcome in terms of clear or positive margin from primary resection, and functional outcome in terms of voice and swallowing were extracted for both transoral robotic and non-Robotic transoral microscopic surgery. These characteristics of the included studies are presented in Tables 1-2.

**Table 1:** Reviewed studies utilizing TORS TORS: transoral robotic surgery

						No of patients with					
	Authors	Year	Study type	Sample size	Mean age	Clear margins	Positive margins	Good voice	Poor voice	Good swallowing	Poor swallowing
1	Sano et al. [18]	2021	Retrospective cohort	57	68	51	5	Not reported	Not reported	Not reported	Not reported
2	Olsen et al. [1]	2013	Retrospective cohort	18	60.6	11	7	18	0	18	0
3	Graboyes et al. [19]	2015	Retrospective cohort	65	53.5	61	4	65	0	65	0
4	Zevallos et al. [20]	2016	Retrospective cohort	369	58	83.20	16.80	Not reported	Not reported	Not reported	Not reported
5	Parhar et al. [21]	2021	Retrospective cohort	56	62	45	11	56	0	53	3
6	Sumer et al. [22]	2013	Retrospective cohort	17	57	15	2	17	0	15	2
7	Moore et al. [23]	2009	Prospective case series	45	57	45	0	45	0	40	5
8	Richmon et al. [24]	2014	Retrospective cohort	116	59.3	Not reported	Not reported	116	0	116	0

**Table 2:** Reviewed studies involving non-TORS TORS: transoral robotic surgery

						No of Patients with					
	Authors	Year	Study type	Sample size	Mean age	Clear margins	Positive margins	Good voice	Poor voice	Good swallowing	Poor swallowing
1	Sano et al. [18]	2021	Retrospective cohort	73	68	53	18	Not reported	Not reported	Not reported	Not reported
2	Zevallos et al. [20]	2016	Retrospective cohort	145	58	71.7%	28.7%	Not reported	Not reported	Not reported	Not reported
3	Sumer et al. [22]	2013	Retrospective cohort	16	57	14	2	16	0	16	0
4	Zoysa et al. [25]	2017	Case study	1	NA	1	0	1	0	1	0
5	Jackson et al. [26]	2021	Retrospective case series	56	59.3	55	1	56	0	51	5

The term “clear margin” was used to describe the complete resection of the tumor from the primary procedure without a specific millimeter measurement.

Voice and deglutition outcomes are graded as good or poor. For the voice, those who did not require a long-term tracheostomy were considered to have a good voice outcome, while those who did require a long-term tracheostomy were considered to have a poor voice outcome, keeping in mind that the tracheostomy was done as a complication of the procedure and not because of the primary disease. Regarding swallowing, those who required a long-term percutaneous endoscopic gastrostomy (PEG) tube were classified as having a poor swallowing outcome, whereas those who did not require a PEG tube were classified as having a good swallowing outcome. Not all eligible studies reported oncologic outcomes, and some studies did not report functional outcomes.

Data Analysis

Of the 10 eligible studies in this review, three studies have data that report both transoral laser microscopic technique and TORS. There were two studies that reported data only on the transoral non-robotic surgery, while there were five studies that reported data only from the transoral robotic approach. Reporting of patient baseline characteristics varied significantly. The total number of patients from all eligible studies was 1034, 743 for robotic transoral surgery, and 291 for non-robotic transoral microsurgery. All the studies were either retrospective analyses or cohort studies, and there were no randomized clinical trial studies. Meta-analysis was not possible to perform because of the variability of study characteristics. Therefore, descriptive analysis was carried out to review the available data.

Results

**Figure 1:**
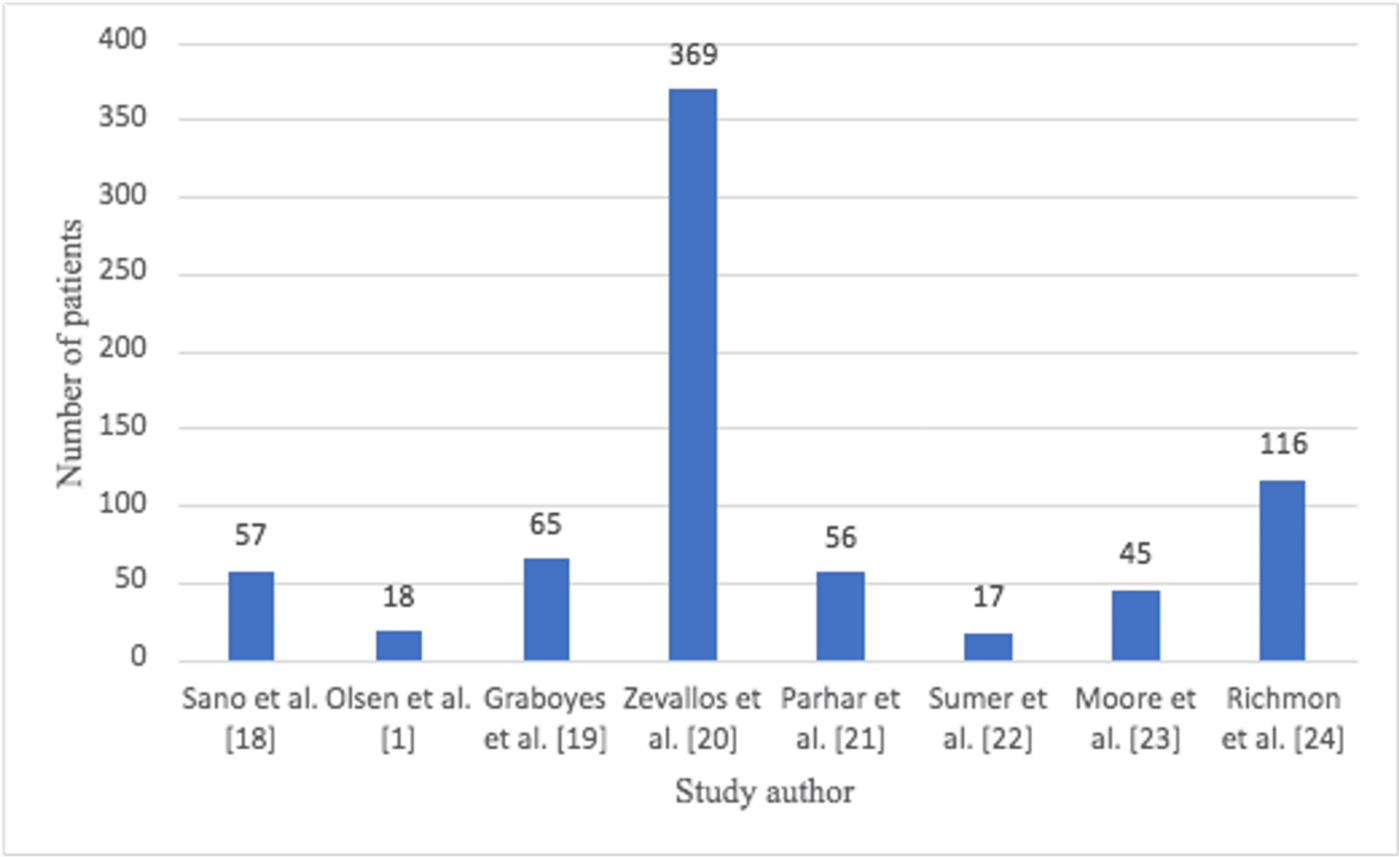
Number of TORS patients in each study TORS: transoral robotic surgery

The total number of patients in TORS studies was 743 from eight studies; however, there was a large variation in sample size between studies (Figure 1).

**Figure 2:**
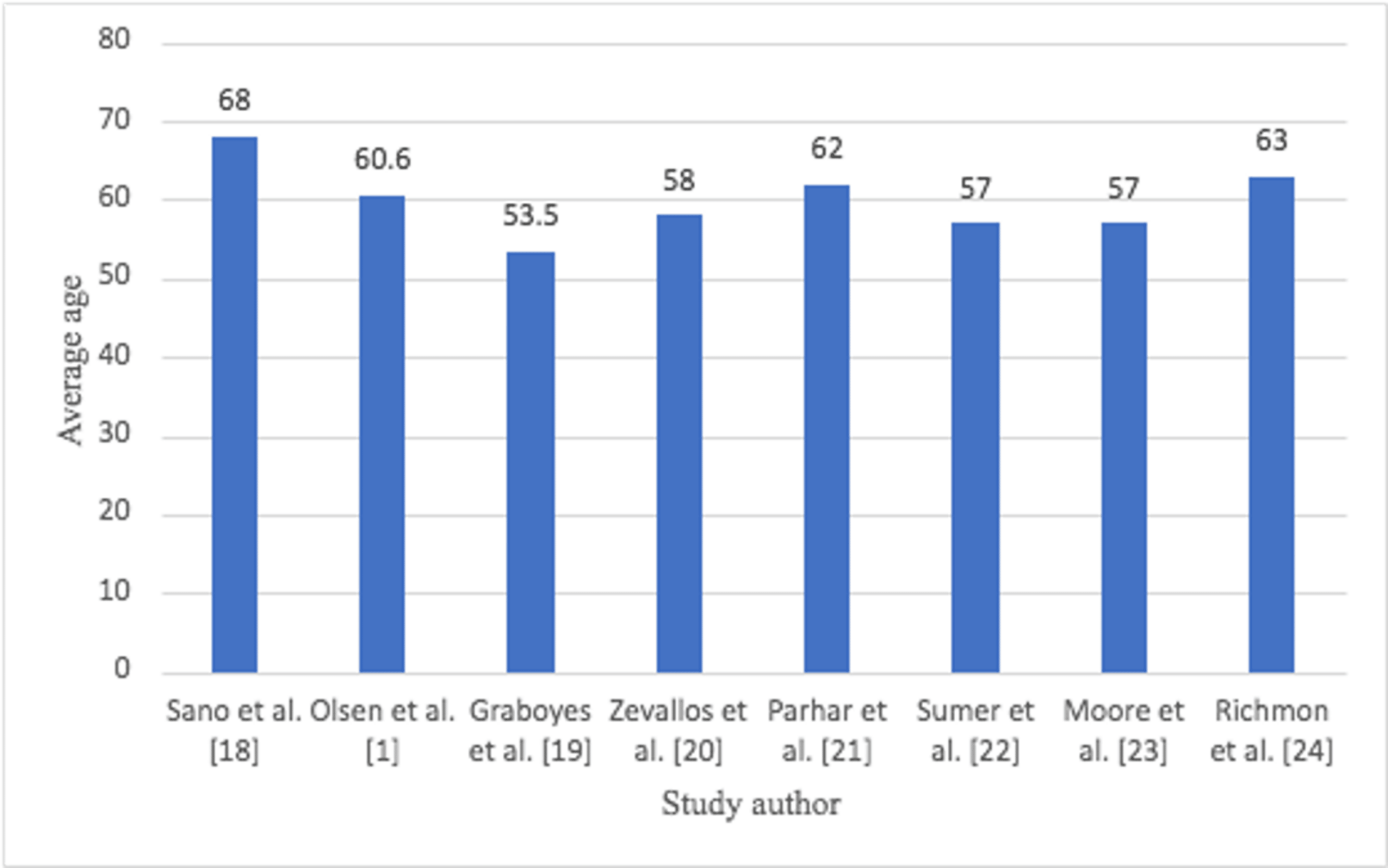
Average age of TORS patients in each study TORS: transoral robotic surgery

The average age for all studies ranged between 60 and 68 years old (Figure 2).

**Figure 3:**
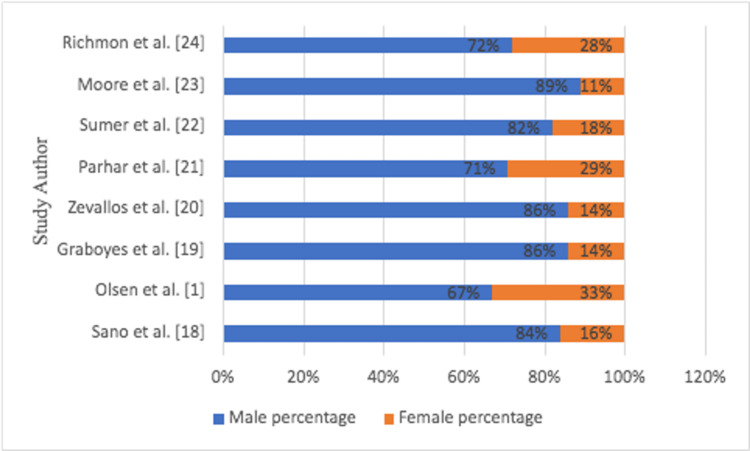
Gender ratio of TORS patients in each study TORS: transoral robotic surgery

In all studies, the male frequency was about 80%, while the female percentage was 20% (Figure 3).

In all TORS studies, the total percentage of patients with clear margins was 72%, while the percentage of patients with positive margins or who required revision surgery was 13%. Some studies, such as Richmon et al, did not report the margins outcome, while others did not report the functional outcomes. Patients who underwent TORS all had successful voice outcomes.

The presence of a long-term tracheostomy or difficulty vocalizing due to tongue resection determined whether the voice outcome was good or poor. There were no patients in the TORS arm who required long-term tracheostomy; in a few patients, tracheostomy was performed in the first two weeks to secure the airway from laryngeal edema, but decannulation was successfully achieved in two to three weeks. In terms of swallowing, the criteria used to determine whether the outcome is good or bad is whether the patient has a long-standing NG tube or a PIG tube; however, patients who had the PIG before the procedure were not involved. The percentage of patients with a good swallowing outcome was 92%, but in Sano et al. and Zevallos et al., the swallowing outcome was not reported [18,20].

**Figure 4:**
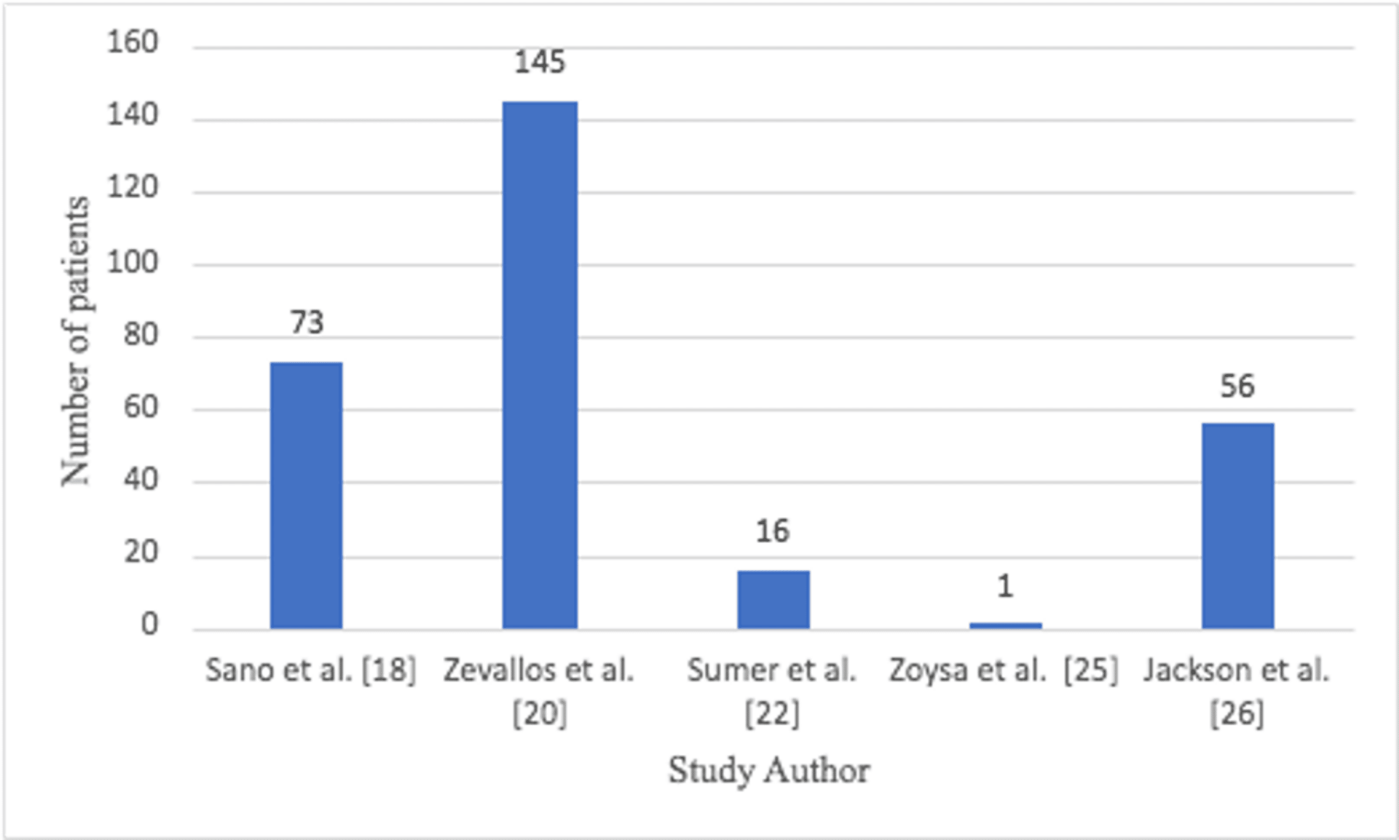
Number of non-TORS patients in each study TORS: transoral robotic surgery

There were 291 patients in the non-TORS group (Figure 4).

**Figure 5:**
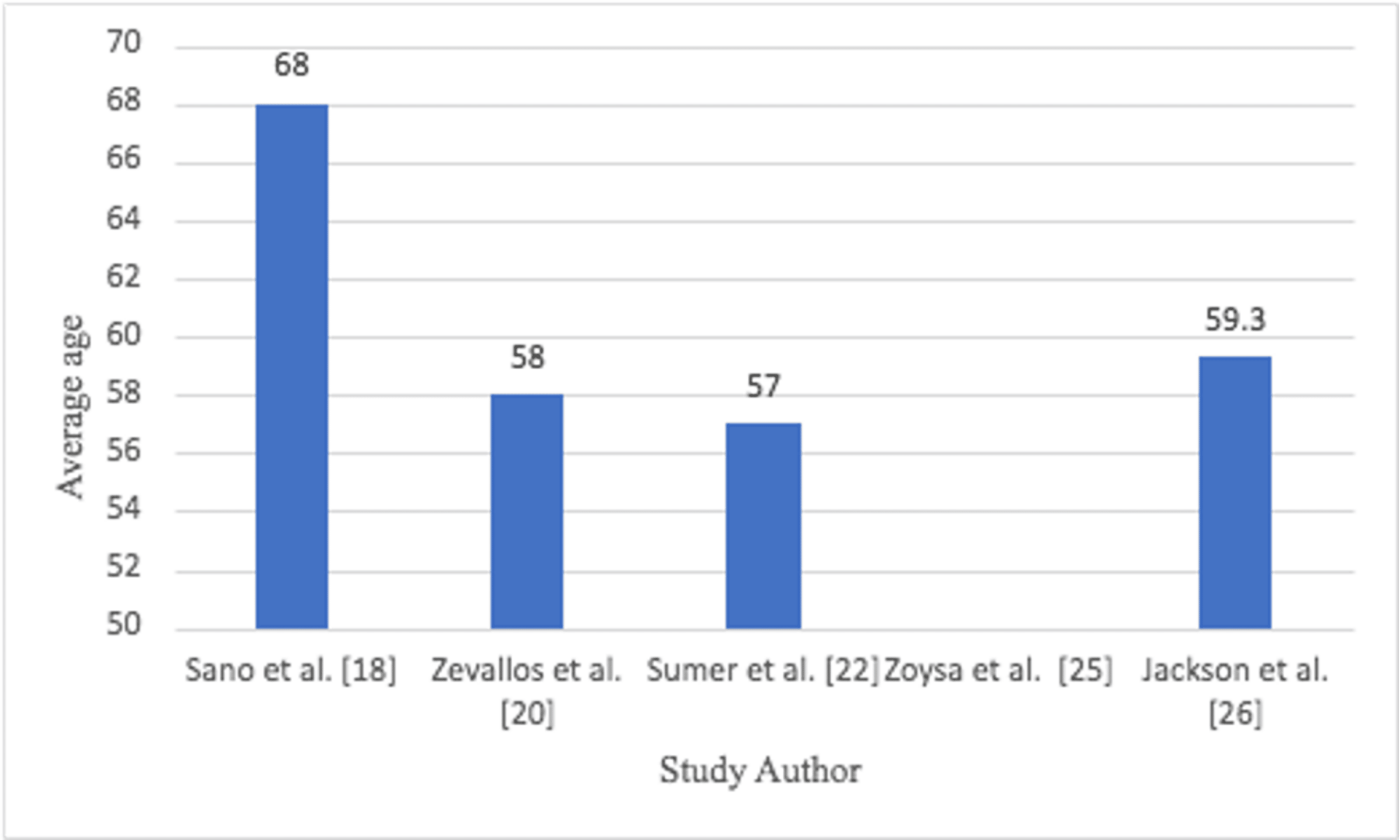
Average age of TORS patients in each study TORS: transoral robotic surgery

The average age ranged between 55 and 65 (Figure 5).

**Figure 6:**
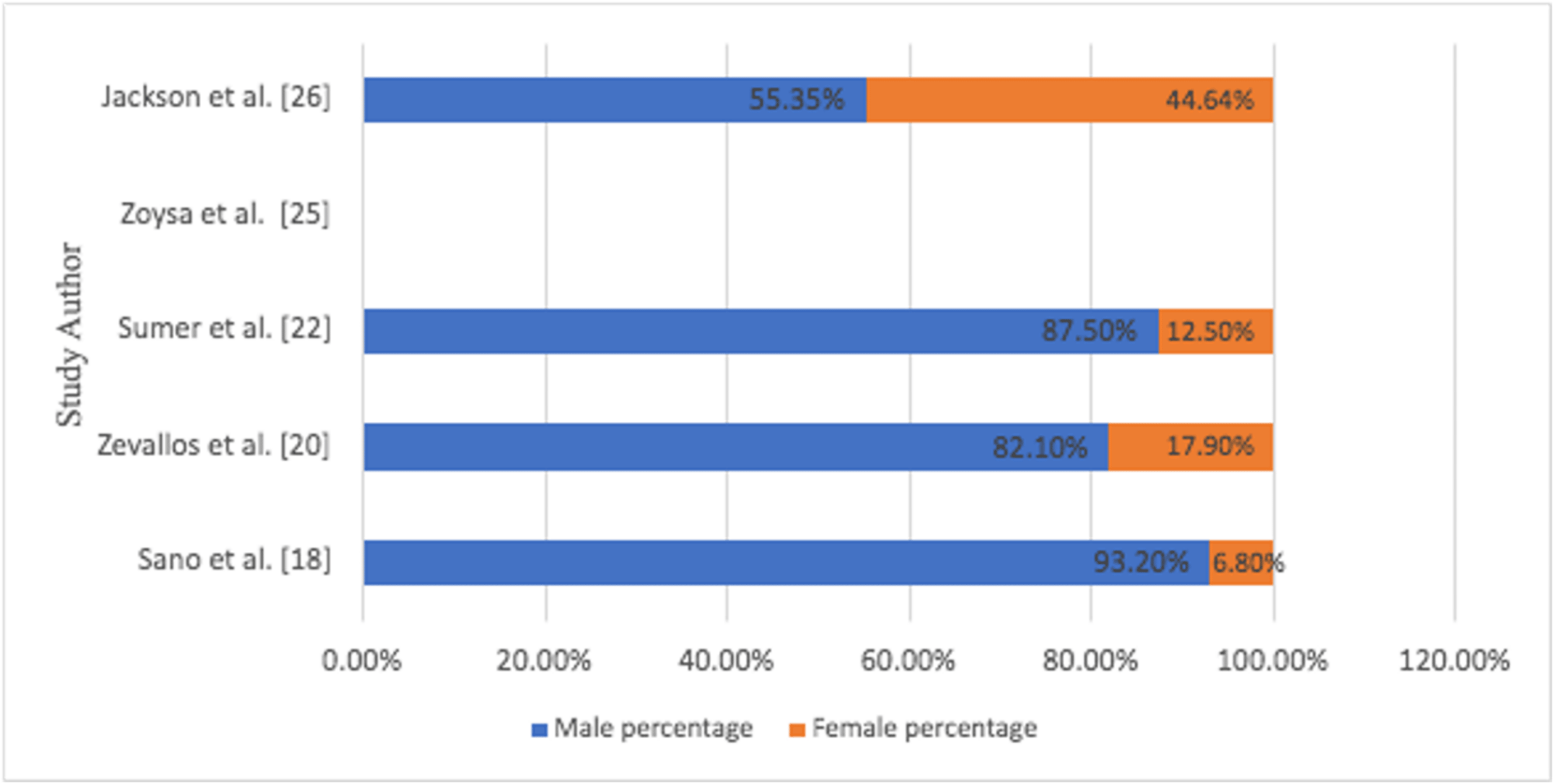
Gender ratio of non-TORS patients in each study TORS: transoral robotic surgery

The gender demographics were like the TORS group, with approximately 80% male and 20% female (Figure 6).

**Figure 7:**
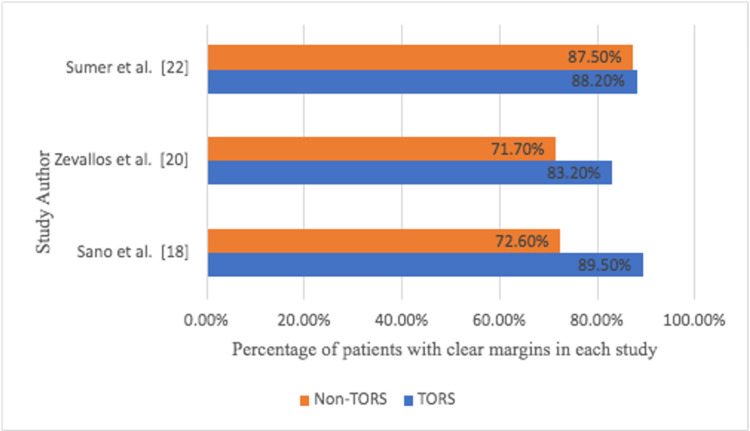
Percentage of patients with clear margins in each study comparing TORS vs non-TORS groups TORS: transoral robotic surgery

**Figure 8:**
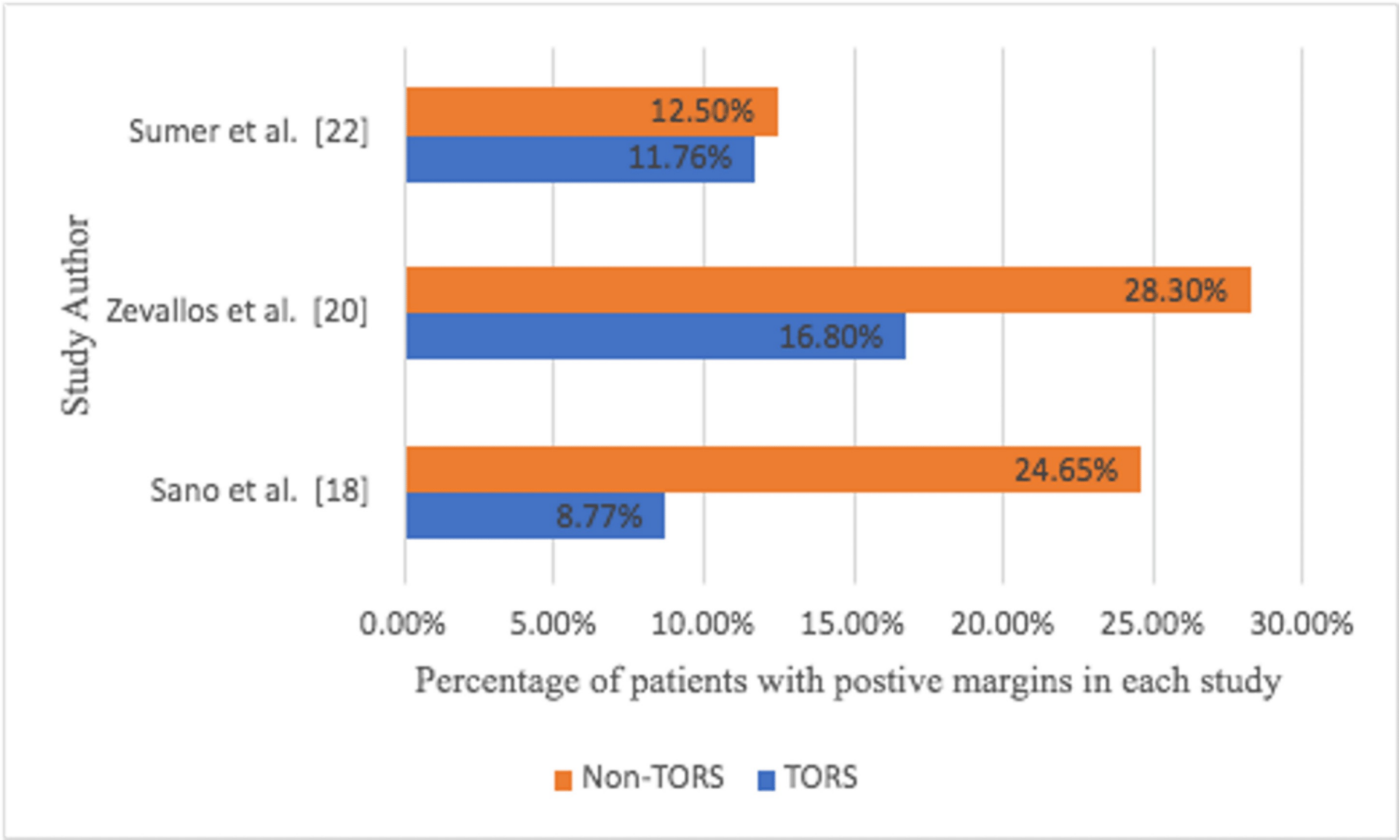
Percentage of patients with positive margins in each study comparing TORS vs non-TORS groups TORS: transoral robotic surgery

The proportion of patients with clear margins with non-TORS was 77.66%, while the proportion of people who were positive was 23.3% (Figures 7-8).

The same criteria were applied to the voice and swallowing outcomes, and there were 100% who had good voice outcomes, while there were 93% who had good swallowing outcomes. Sano et al. and Zevallos et al., on the other hand, did not report the functional outcome also [18,20].

Taking all TORS as one sample and all non-TORS as one sample, non-TORS achieves clear margins about 4% better than TORS, but this discrepancy between results when considering the three comparison studies can be explained by the large variation in sample size between the two techniques [19]. When TORS is used as the control sample size for non-TORS, statistically, TORS is 30% better at achieving clear margins [20].

There were three studies that compared the two methods, but the data was retrospective analysis rather than RCTs, so descriptive charts were created. The total number of patients in these studies who were treated with TORS was 443, while the number of patients treated without TORS was 234 with similar mean age and gender distribution percentages [21]. Sumer et al. found that TORS achieved 88.2% clear margins while non-TORS achieved 87.5 [22]. In Zevallos et al.’s study, patients who had TORS had 83.2% clear margins compared to 71.70% clear margins in patients who did not have TORS [20]. In Sano et al.’s study, 89.5% of the patients had clear margins, while 72.6 had clear margins with non-TORS [18]. When the two techniques are compared, the TORS is statistically better at achieving clear margins [22,23].

In summary, in Zevallos et al. and Sano et al.’s papers, positive margins for TORS were 11.76%, 16.80%, and 8.77%, respectively while non-TORS margins were 12.50%, 28.30%, and 24.65%, respectively. As a result, the percentage clear margin difference between TORS and non-TORS in Sumer, Zevallos, and Sano’s papers is 0.7%, 11.5%, and 16.9%, respectively. TORS, on the other hand, had lower positive margins with the same percentage as non-TORS [24].

Discussion

OPSCC is on the rise and will continue to rise [25,27]. Aside from the traditional risk factors of chronic smoking and alcohol consumption, the human papillomavirus (HPV) has emerged as the leading cause of OPSCC in the United Kingdom and other countries. Until recently, there was no consensus on the best treatment strategies for patients with OPSCC [11]. Prior to the last two decades, the most frequently used strategies were primary surgery and primary radiotherapy. However, recent organ preservation trials have shown that CRT outperforms radiotherapy alone in terms of survival, leading to increased use of CRT [26]. On the other hand, there has recently been a resurgence of interest in using primary surgical approaches to treat oropharyngeal SCC, especially because of the rising incidence of HPV-related OPSCC, therefore, the need for tailoring treatment with TORS or TLM is imminent.

TLM was initially used and produced satisfactory results, but due to poor visualization of the entire surgical field provided by the microscope and the low hemostatic efficacy of laser, it was necessary to alternate with classical hemostasis techniques and TORS was the option. TORS is becoming more popular in head and neck surgery, as well as in oropharyngeal carcinoma, particularly in early-stage disease. However, setting up a robot to carry out surgeries is costly and requires special training for surgeons and theater staff, compared to the TLM, which only requires a microscope that is always available in the ENT theater and does not require much training. As a result, this study is being conducted to evaluate the oncological and functional outcomes to standardize the surgical treatment for early oropharyngeal SCC. When comparing overall oncological findings, TORS has better clear margins than non-TORS, though this can only be seen in three studies where both techniques were compared retrospectively. In Sano et al.’s study, TORS improved the percentage of clear margins by 16.9%. Sano’s study was a multi-center study that compared TORS treatment outcomes to non-robotic transoral surgeries. TORS was shown to have a lower likelihood of positive margins than non-robotic surgeries. In the subgroup analysis of patients with OPSCC, the TORS cohort had significantly fewer patients with surgical positive margins than the non-robotic cohorts. These findings indicate that TORS is a viable surgical strategy for ensuring tumor resection with negative surgical margins in patients with early-stage laryngopharyngeal cancer. Zevallos et al. also discovered that TORS outperforms non-TORS by 11.5% in terms of achieving clear margins. In his study and to better understand how transoral endoscopic surgery is used in routine clinical care, early patterns of care for patients with oropharyngeal SCC were examined in a large national cancer center. Furthermore, short-term surgical outcomes were evaluated with a focus on surgical margin status to assess the generalizability of previously reported patient surgical outcomes in the literature and to establish baseline outcomes observed during the early adoption of TORS and non-TORS. Transoral endoscopic resection for OPSCC appears to be well tolerated, with small numbers of unplanned hospital readmissions or perioperative mortality observed. In Sumer et al.’s study, hospital stay, blood loss, and fluids administered were comparable for both groups of patients. There were no statistically significant differences in functional outcomes between the two techniques (TORS is better by 0.7% than non-TORS), and there were no significant differences in the need for tracheostomy or gastrostomy tubes. Most patients were able to resume their normal diet immediately after their transoral surgery. The follow-up time for the cohort of patients in the same study was too short, and the number of events was too small for meaningful comparison of oncologic outcomes. However, both techniques have demonstrated excellent When all TORS papers are compared to non-TORS papers, it is found that TORS has better oncological outcomes. This is understandable because TORS has advantages such as increased visualization, magnification, and stabilized robotic arms, which may contribute to surgical resection adequacy and thus reduce the risk of postoperative RT or CRT, and the ability to achieve negative margins, the most important surgical variable affecting loco-regional control. In terms of long-term functional outcome, there is no discernible difference between the two methods; however, the total number of TORS arms is greater than the total number of non-TORS arms. The advantages of TORS versus non-robotic endoscopic surgical approaches for oropharyngeal SCC are debatable. TORS, according to the authors of the papers, provides better visualization and exposure for oropharyngeal SCC than non-robotic transoral approaches. Although both TORS and non-robotic transoral are limited by a lack of haptic feedback, proponents of TORS point to the ability to maneuver with two hands and overcome line-of-sight constraints as potential advantages of TORS over TLM.

Limitations

This study has a few limitations. This includes the lack of RCT to incorporate into the data analysis. Furthermore, because some of the studies are retrospective in nature, there is a risk of incorrectly or incompletely coded data. Furthermore, the outcomes measurements were not reported consistently across all studies, affecting the overall sample size of the measurement. Some of the studies chosen had selection biases. There was also a publication bias because only English texts were chosen. However, there was a discrepancy in results when comparing two techniques as one sample, which can be explained by the large variation in sample size between the two techniques. To overcome this disparity, narrative analysis was used. Because of the scarcity of data, more prospective studies and RCTs are required.

## Conclusions

One of the most common types of head and neck cancer is OPSCC. Recently, there has been an increase in interest in surgical treatment for early stages, but whether this would have an impact on the oncological outcome and functional outcome was the question that this study attempted to answer. When comparing TORS to non-TORS, TORS has a better oncological and functional outcome. These findings emphasize the importance of surgeon experience and patient selection in achieving better patient outcomes. Because TORS is a new technique, it is recommended that it be included in the surgeon training curriculum. Clinical trials focusing on transoral surgery for HPV-positive or negative patients will aid in the further definition of indications, functional outcomes, and oncologic outcomes associated with transoral surgery for OPSCC.
